# Identification of Cellular Isoschaftoside-Mediated Anti-Senescence Mechanism in *RAC2* and *LINC00294*

**DOI:** 10.3390/molecules29174182

**Published:** 2024-09-04

**Authors:** Yun Haeng Lee, Byeong Hyeon So, Kyeong Seon Lee, Myeong Uk Kuk, Ji Ho Park, Jee Hee Yoon, Yoo Jin Lee, Du Yeol Kim, Min Seon Kim, Hyung Wook Kwon, Youngjoo Byun, Ki Yong Lee, Joon Tae Park

**Affiliations:** 1 Division of Life Sciences, College of Life Sciences and Bioengineering, Incheon National University, Incheon 22012, Republic of Korea; yh.lee@inu.ac.kr (Y.H.L.); tundra@inu.ac.kr (B.H.S.); muk20@inu.ac.kr (M.U.K.); 202428002@inu.ac.kr (J.H.P.); yoojn0905@inu.ac.kr (J.H.Y.); juli9709@inu.ac.kr (Y.J.L.); papaya1130@inu.ac.kr (D.Y.K.); alstjs0323@inu.ac.kr (M.S.K.); hwkwon@inu.ac.kr (H.W.K.); 2 College of Pharmacy, Korea University, Sejong 30019, Republic of Korea; kslee0118@korea.ac.kr (K.S.L.); yjbyun1@korea.ac.kr (Y.B.); 3 Convergence Research Center for Insect Vectors, Incheon National University, Incheon 22012, Republic of Korea

**Keywords:** senescence amelioration, flavonoid, isoschaftoside, reactive oxygen species (ROS)

## Abstract

As cellular senescence, reactive oxygen species (ROS) accumulate excessively, causing cellular damage. Flavonoids derived from natural products are known for their antioxidant effects and their ability to delay cellular senescence. Previous studies have attempted to mitigate cellular senescence using flavonoids from natural sources. However, the detailed mechanisms and regulatory targets of some flavonoids exhibiting antioxidant effects have not been fully elucidated. Therefore, we screened a library of flavonoids for antioxidant properties. Isoschaftoside, a glycosidic flavonoid, significantly reduced ROS levels in senescent cells. It was found that mitochondrial function was restored, and dependence on glycolysis was reduced in senescent cells treated with isoschaftoside. Additionally, we identified that isoschaftoside suppresses ROS by reducing the expression of *RAC2* and *LINC00294* in senescent cells. Taken together, this study establishes a novel mechanism for ROS inhibition and the regulation of cellular senescence by isoschaftoside. Our findings contribute important insights to antioxidant and anti-senescence research.

## 1. Introduction

The skin consists of three layers (the epidermis, dermis, and subcutaneous fat) and is an epithelial tissue that covers our body [1]. It acts as a barrier to the environment, protects against microorganisms, and maintains body fluids and temperature [1]. Skin aging is the process by which the quality of the cells that make up skin tissue deteriorates with age. This process leads to the thinning of the epidermis and the flattening of the dermal–epidermal junction [2]. As cells age, epidermal cell turnover decreases, resulting in poorer exfoliation and slower wound healing [2]. The dermis undergoes the most significant microstructural changes during aging. Deterioration in the dermis involves the separation of the dermis and epidermis, leading to looser skin and a reduced regeneration of epidermal stem cells [2]. Fibroblasts, the most common cells in the dermis, decrease in both number and function with age [2]. Skin aging changes the function of cellular organelles, especially with regard to mitochondrial degeneration [3]. As skin ages, defective mitochondria accumulate and undergo structural changes that significantly increase mitochondrial volume and size [3]. Strategies are needed to restore the functionality of structurally and functionally aged skin.

Strategies to slow skin aging include a variety of mechanisms and therapeutic approaches [4]. First, sunscreen use is an important way to prevent skin aging by preventing DNA damage and collagen degradation caused by ultraviolet (UV) radiation exposure [5]. Second, retinoids are effective in reducing wrinkles and improving skin elasticity by promoting skin cell turnover and stimulating collagen production [6]. Additionally, treatments such as skin moisturizers can maintain the skin’s moisture barrier to prevent dryness and loss of elasticity [7]. In intracellular mechanisms, oxidative stress targets defective mitochondria, which further enhances mitochondrial reactive oxygen species (ROS) production, in addition to ROS production [8]. Increased oxidative stress caused by ROS leads to protein oxidation, lipid peroxidation, and the chain scission of collagen and elastin, worsening skin aging [9]. Therefore, antioxidant strategies that reduce ROS production may be effective as a therapeutic approach for skin aging.

As part of cellular senescence, defective mitochondria accumulate and undergo structural changes that significantly increase their volume and size [10]. These dysfunctional mitochondria leak electrons from complexes I and III of the electron transport chain (ETC), generating ROS as a byproduct [11,12]. ROS are generally classified into superoxide anions (^●−^O_2_), hydroxyl radicals (^●^OH), and hydrogen peroxide (H_2_O_2_) [13]. In addition to ROS production, defective mitochondria are targets of oxidative stress [14]. As a result, more ROS are generated from dysfunctional mitochondria, accelerating senescence [14]. The accumulation of ROS can cause inflammation and DNA damage in cells. In other words, excessive ROS can cause permanent damage to cells and accelerate senescence [14]. Therefore, strategies to reduce ROS accumulation in cells require extensive research to improve senescence outcomes.

Secondary metabolites derived from natural products are widely used in modern medicine and daily life [15]. Cosmetics development is no exception [16,17]. Secondary metabolites derived from natural products are already being studied in cosmetics development [16,17]. Flavonoids are secondary metabolites found in various plants and are known as natural polyphenols [18,19]. They have a basic flavone skeleton consisting of two benzene rings and a pyran ring connecting them [18]. The structural diversity of flavonoids is mainly achieved by various substituents such as hydroxyl groups (–OH), methoxy groups (–OCH_3_), and sugar residues (glycosyl) [18]. Most flavonoid compounds exist in the form of glycosides, where the sugar moiety is linked to a phenolic hydroxyl group [20]. Several previous studies have shown that flavonoids exert antioxidant and anti-inflammatory effects [21]. Representative polyphenols with these effects include the epigallocatechin gallate as a flavonoid and the resveratrol as a stilbene [21]. Epigallocatechin gallate, primarily found in green tea, has powerful antioxidant and anti-inflammatory properties [21]. Resveratrol, first discovered in grapes, has been reported to improve cardiovascular health and help prevent aging, in addition to its antioxidant and anti-inflammatory effects [22]. However, the antioxidant and senescence rejuvenation mechanisms of some natural products have not yet been fully elucidated.

In this study, we aimed to identify antioxidants and mechanisms that effectively inhibit ROS by screening an astragalin isolated from *Orostachys japonicus (O. japonicus*), isoschaftoside isolated from *Viola yedoensis* (*V. yedoensis*), skullcapflavone II isolated from *Scutellaria baicalensis* (*S. baicalensis*), and 2′-hydroxygenistein isolated from *Apios americana* (*A. americana*). Among the flavonoids, isoschaftoside effectively reduced ROS and improved cell metabolism. Additionally, RNA expression analysis showed that isoschaftoside downregulates *RAC2* and *LINC00294*. Here, we define the anti-senescence and anti-inflammatory mechanisms of isoschaftoside and propose a downregulation strategy.

## 2. Results

### 2.1. Isoschaftoside Significantly Reduces ROS in Senescent Cells

**Figure 1:**
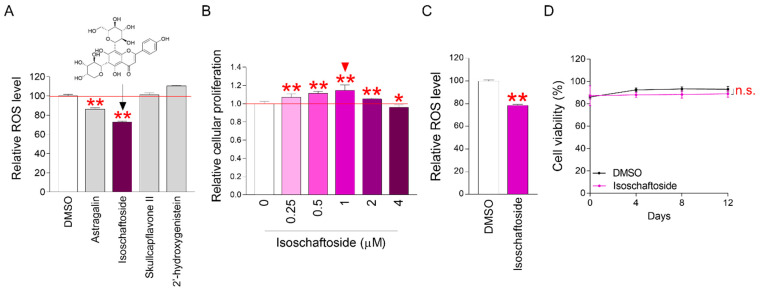
Optimal concentrations of isoschaftoside significantly reduce reactive oxygen species (ROS) in senescent cells. (**A**) Senescent cells were treated with astragalin, isoschaftoside, skullcapflavone Ⅱ, and 2′-hydroxygenistein at a concentration of 4 μM each for 12 days (detailed reagent information is listed in Table 1). Then, senescent cells were stained with DHR123 at a concentration of 10 μg/mL, and ROS was measured by FACS. Astraglin and isoschaftoside were confirmed to significantly reduce ROS. ** *p* < 0.01, Student’s *t*-test. Mean ± SD, n = 3. (**B**) Cell proliferation assay was performed to optimize the concentration of isoschaftoside that reduces ROS the most. Cell proliferation assay was performed using DNA content assay. It significantly increased cell proliferation of senescent cells at a concentration of 1 μM (red triangle is the optimized concentration). Subsequent studies were performed at the selected concentration. * *p* < 0.05, ** *p* < 0.01, Student’s *t*-test. Mean ± SD, n = 6. (**C**) The ROS-reducing effect of isoschaftoside was also confirmed at the optimized concentration (1 μM). ** *p* < 0.01, Student’s *t*-test. Mean ± SD, n = 3. (**D**) Isoschaftoside at the optimized concentration did not show toxicity in senescent cells. Cell viability was measured by a Cedex HiRes Analyzer. n.s. (not significant) two-way ANOVA followed by Bonferroni post hoc test. Mean ± SD, n = 3.

Previous studies have shown that astragalin, isoschaftoside, skullcapflavone II, and 2′-hydroxygenistein have anti-inflammatory and some antioxidant activities [23,24,25,26,27,28,29]. Antioxidant activity is the action of removing ROS, which improves senescence [30]. It is also well known that senescence is improved by reducing the inflammatory phenotype [31]. Therefore, we used fluorescence-activated cell sorting (FACS) to identify candidates that could inhibit ROS production in senescent cells using flavonoids that have been shown to be effective in previous studies (Table 1). The final concentration for library screening was 4 μM. Skullcapflavone II did not cause noticeable ROS changes compared to DMSO (negative control) (Figure 1A). An increase in ROS was observed with 2′-hydroxygenistein compared to DMSO (Figure 1A). On the other hand, a significant reduction in ROS was observed with astragalin and isoschaftoside compared to DMSO (Figure 1A). These results suggest that astragalin and isoschaftoside have the potential to restore mitochondrial function by reducing ROS in senescent cells.

In this study, isoschaftoside showed the most significant ROS recovery effect. Therefore, we screened the flavonoid library and selected isoschaftoside as a candidate for improving senescence. Isoschaftoside concentration optimization was performed for further experiments. Cellular senescence induces cell-cycle arrest and reduces cell proliferation. Therefore, cell proliferation was measured depending on the concentration of isoschaftoside. The isoschaftoside concentration range was 0.25–4 μM. The increase in cell proliferation rate was most significant at a concentration of 1 μM (Figure 1B). These data suggest that 1 μM isoschaftoside restores cell-cycle function in senescent cells.

Concentration optimization was performed for isoschaftoside, and 1 μM was chosen. To determine whether 1 μM also had an ROS inhibition effect, we measured ROS using FACS. Isoschaftoside treated at 1 μM significantly reduced ROS concentration in senescent cells (Figure 1C). These findings indicate that the ROS-reducing effect of isoschaftoside is reproduced even at optimized concentrations. Isoschaftoside was found to be effective in reducing ROS in senescent cells. However, the cytotoxicity of isoschaftoside also needed to be assessed. After treating senescent cells with 1 μM isoschaftoside, cell viability was monitored for 12 days. Over this period, isoschaftoside had no significant effect on cell viability (Figure 1D). These data suggest that 1 μM isoschaftoside is not cytotoxic to senescent cells. Taken together, these results suggest that the flavonoid isoschaftoside is effective in improving senescence functions.

### 2.2. Isoschaftoside Restores Mitochondrial Function and Reduces Glycolysis Dependence

**Figure 2:**
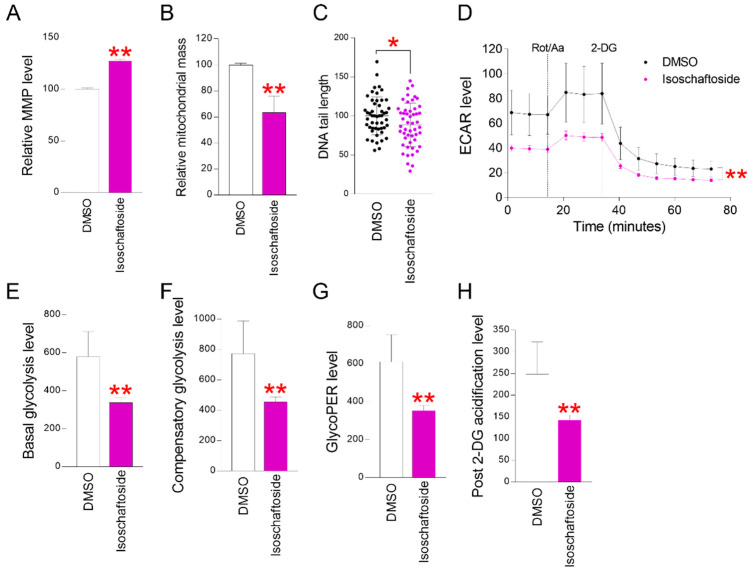
Isoschaftoside restored cellular metabolism by improving mitochondria. (**A**) Senescent cells treated with isoschaftoside were stained with JC-10 and analyzed by FACS. Isoschaftoside significantly restored mitochondrial membrane potential (MMP). ** *p* < 0.01, Student’s *t*-test. Mean ± SD, n = 3. (**B**) Senescent cells treated with isoschaftoside were stained with Mitotracker deep red and analyzed by FACS. Isoschaftoside significantly reduced mitochondrial mass. ** *p* < 0.01, Student’s *t*-test. Mean ± SD, n = 3. (**C**) DNA damage analysis of isoschaftoside-treated senescent cells was performed using comet assay. DNA was stained with GelGreen reagent and imaged by fluorescence microscopy at 488 nm/535 nm. DNA tail length was measured using the image j tool. The dots represent the length of each DNA tail. * *p* < 0.05, Student’s *t*-test. Mean ± SD, n = 40. (**D**) Extracellular acidification rate (ECAR) of isoschaftoside-treated senescent cells was measured using a Seahorse apparatus (port A: rotenone/antimycin; Rot/Aa, port B: 2-deoxyglucose; 2-DG). Isoschaftoside-treated senescent cells significantly reduced ECAR. ** *p* < 0.01, two-way ANOVA followed by Bonferroni post hoc test. Mean ± SD, n = 3. (**E**) Basal glycolysis rate measured by ECAR. Isoschaftoside-treated senescent cells significantly reduced basal glycolysis levels. ** *p* < 0.01, Student’s *t*-test. Mean ± SD, n = 3. (**F**) After Rot/Aa injection: (maximal increase in acidification rate)—(base acidification rate) value. This value represents the potential glycolytic capacity. Senescent cells treated with isoschaftoside showed a significant decrease in compensatory glycolysis levels. ** *p* < 0.01, Student’s *t*-test. Mean ± SD, n = 3. (**G**) The graph compares the glyco proton efflux rate (PER) values between DMSO and isoschaftoside. Senescent cells treated with isoschaftoside showed a significant decrease in glycoPER levels. ** *p* < 0.01, Student’s *t*-test. Mean ± SD, n = 3. (**H**) Minimal decrease in acidification rate after 2-deoxyglucose (2-DG) treatment. This value represents the activation of metabolic pathways other than mitochondrial. Senescent cells that were treated with isoschaftoside exhibited a notable decrease in post-2-DG acidification levels. ** *p* < 0.01, Student’s *t*-test. Mean ± SD, n = 3.

Dysfunction of the mitochondrial ETC reduces the H^+^ concentration gradient in the inner membrane space [12]. This dysfunction accelerates ROS production and causes further mitochondrial impairment [12]. In this study, isoschaftoside effectively inhibited ROS in senescent cells. Therefore, we examined whether mitochondrial function was restored as a result of ROS inhibition. Mitochondrial membrane potential (MMP) was determined using fluorescence-activated cell sorting (FACS). Isoschaftoside significantly increased MMP in senescent cells (Figure 2A). The increase in MMP suggests the restoration of mitochondrial function in senescent fibroblasts. Previous studies have reported that mitochondrial mass increases when mitochondrial function declines due to senescence [32]. Therefore, we investigated whether isoschaftoside-mediated MMP recovery is linked to changes in mitochondrial mass. A significant decrease in mitochondrial mass was observed in isoschaftoside-treated senescent cells (Figure 2B). These findings suggest that isoschaftoside restores mitochondrial function in senescent cells.

ROS generated by cellular senescence mainly damage DNA, and DNA damage can be used as an indicator of cellular senescence improvement [33]. Comet assays were performed to determine the presence of DNA damage. The DNA tail length of senescent cells was significantly reduced as a result of isoschaftoside treatment (Figure 2C). These data show that isoschaftoside reduces DNA damage and fragmentation.

As cells undergo senescence, mitochondrial function declines, and glycolysis is primarily used to generate intracellular energy [32]. The increased reliance on glycolysis in senescent cells disrupts intracellular metabolic balance [34]. Given that mitochondrial function was restored, it was necessary to confirm the normalization of glycolysis. Therefore, we investigated the regulation of glycolysis in senescent cells mediated by isoschaftoside. The glycolysis rate was measured as the extracellular acidification rate (ECAR). Isoschaftoside reduced overall ECAR in senescent cells (Figure 2D). These data suggest that isoschaftoside reduces the rate of glycolysis in senescent cells. Additionally, both basal and compensatory glycolysis were reduced (Figure 2E,F). The reduction in basal and compensatory glycolysis supports the observed decrease in glycolysis rates due to isoschaftoside treatment.

After glycolysis occurs within the cell, protons not used for cellular respiration are used to produce lactate [35]. Lactate fermentation is an inefficient energy metabolic process that produces only a small amount of energy [35]. As reliance on glycolysis increases, more protons are produced and released as pyruvate is converted to lactate [36]. Therefore, we calculated the proton efflux rate (GlycoPER) of glycolysis. A significant decrease in GlycoPER levels was observed in isoschaftoside-treated senescent cells (Figure 2G). These data suggest a decrease in lactic acid production and support a reduction in glycolysis rate. This was further confirmed after acidification with 2-deoxyglucose (2-DG), which includes additional factors contributing to extracellular acidification not related to glycolysis or the mitochondrial tricarboxylic acid (TCA) cycle. A significant reduction in acidification was observed following 2-DG treatment in isoschaftoside-treated senescent cells (Figure 2G). This indicates that intracellular energy metabolic activities are shifted towards the TCA cycle through isoschaftoside mediation. Taken together, these results suggest that isoschaftoside induces metabolic changes in senescent cells that restore mitochondrial function and reduce dependence on glycolysis.

### 2.3. Isoschaftoside Activates the Autophagy System to Clear Senescent Cells

**Figure 3:**
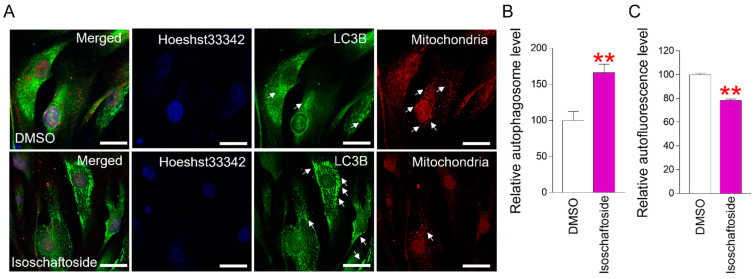
Isoschaftoside restores the autophagy system in senescent cells. (**A**) Autophagosomes of DMSO- and isoschaftoside-treated senescent cells were visualized using immunofluorescence (blue: Hoechst33342, green: anti-LC3B Ab, red: anti-OXPHOS Ab). Isoschaftoside was found to increase LC3B fluorescence and decrease OXPHOS fluorescence in senescent cells (white arrows). The scale bar size is 25 µm. (**B**) Senescent cells treated with DMSO and isoschaftoside were stained with CYTO–ID and analyzed by FACS. Isoschaftoside significantly increased the autophagosome level in senescent cells. ** *p* < 0.01, Student’s *t*-test. Mean ± SD, n = 3. (**C**) Lipofuscin levels were quantified in senescent cells that were exposed to DMSO and isoschaftoside. Lipofuscin was measured by FACS (using FITC channel) as a background green wavelength without staining the cells. Isoschaftoside significantly reduced autofluorescence levels in senescent cells. ** *p* < 0.01, Student’s *t*-test. Mean ± SD, n = 3.

The elimination function of cells is known as the autophagy system [37]. Autophagosome function is known to be reduced in senescent cells [38]. Consequently, senescent cells have a weakened scavenging function, leading to the excessive accumulation of cellular debris [38]. The activation degree of microtubule-associated protein 1A/1B–light chain 3B (LC3B), a membrane protein of autophagosomes, was confirmed using immunofluorescence. LC3B was noticeably activated in isoschaftoside-treated senescent cells (Figure 3A). The mitochondrial retrograde response (MRR) is a signaling mechanism that sends signals from mitochondria to the nucleus [39]. As cellular senescence, dysfunctional mitochondrial and nuclear signaling increases [39]. Therefore, many mitochondria are observed around the nucleus in senescent cells. Perinuclear mitochondria were reduced in isoschaftoside-treated senescent cells (Figure 3A). These data suggest that isoschaftoside activates the autophagy pathway in senescent cells to remove dysfunctional mitochondria.

Additionally, this study used FACS to quantify the extent of autophagosome activation. Isoschaftoside-treated senescent cells showed significantly enhanced levels of autophagy (Figure 3B), supporting the immunofluorescence data indicating autophagosome activation. To determine whether the autophagy system was improved in senescent cells, we identified indicators of intracellular accumulation. Lipofuscin is a polymer found in lysosomes, consisting primarily of protein residues cross-linked due to an oxidation process catalyzed by iron [40]. Due to its non-degradable nature and resistance to exocytosis, the intracellular accumulation of lipofuscin is inevitable after mitosis [40]. In this study, a significant reduction in lipofuscin was observed in isoschaftoside-treated senescent cells (Figure 3C). These data suggest that the decrease in lipofuscin in senescent cells is a result of enhanced intracellular clearance, supporting improvements in the autophagic system.

### 2.4. Isoschaftoside Enhances Characteristics Associated with Skin-Aging Phenotypes

**Figure 4:**
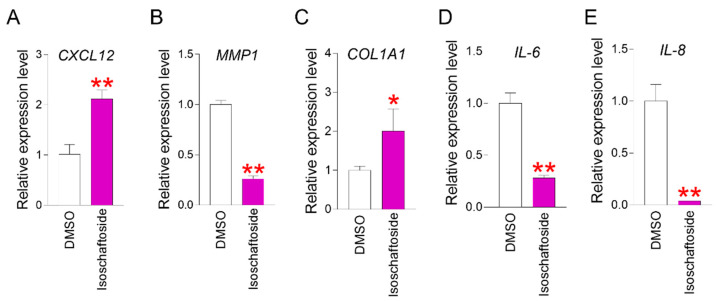
Effect of isoschaftoside on various skin-aging phenotypes. (**A**) After treating senescent cells with isoschaftoside, *CXCL12* gene expression level was measured by qPCR (the qPCR primers used are listed in Table 2). Isoschaftoside significantly increased *CXCL12* in senescent cells. ** *p* < 0.01, Student’s *t*-test. Mean ± SD, n = 3. (**B**) After treating senescent cells with isoschaftoside, *MMP1* gene expression level was measured by qPCR (the qPCR primers used are listed in Table 2). Isoschaftoside significantly decreased *MMP1* in senescent cells. ** *p* < 0.01, Student’s *t*-test. Mean ± SD, n = 3. (**C**) After treating senescent cells with isoschaftoside, *COL1A1* gene expression level was measured by qPCR (the qPCR primers used are listed in Table 2). Isoschaftoside significantly increased *COL1A1* in senescent cells. * *p* < 0.05, Student’s *t*-test. Mean ± SD, n = 3. (**D**) After treating senescent cells with isoschaftoside, *IL-6* gene expression level was measured by qPCR (the qPCR primers used are listed in Table 2). Isoschaftoside significantly decreased *IL-6* in senescent cells. ** *p* < 0.01, Student’s *t*-test. Mean ± SD, n = 3. (**E**) After treatment of senescent cells with isoschaftoside, the level of *IL-8* gene expression was measured by qPCR (the qPCR primers used are listed in Table 1). Isoschaftoside significantly reduced *IL-8* in senescent cells. ** *p* < 0.01, Student’s *t*-test. Mean ± SD, n = 3.

According to previous studies, *C–X–C motif chemokine ligand 12* (*CXCL12*) is known to promote cell migration, such as that of fibroblasts, and plays an important role in skin regeneration [41]. A decrease in *CXCL12* is observed in senescent cells, where skin regeneration is reduced [42]. Therefore, *CXCL12* expression was confirmed in this study. A significant increase in *CXCL12* expression was observed in isoschaftoside-treated senescent cells (Figure 4A). These data suggest that isoschaftoside is effective in promoting skin cell regeneration.

In senescent skin cells, an enzyme called *matrix metalloprotease 1* (*MMP1*) is produced that decomposes collagen [43]. Therefore, the expression level of *MMP1* was examined in this study. The expression of *MMP1* was greatly reduced by isoschaftoside (Figure 4B). Additionally, collagen protein synthesis decreases in cellular senescence, causing the skin to become thinner and lose elasticity, leading to wrinkles and sagging [44]. Consequently, the expression level of *collagen type I alpha 1* (*COL1A1*) was measured. The expression of *COL1A1* was significantly increased in isoschaftoside-treated senescent cells (Figure 4C). This supports data showing decreased collagenase gene expression.

Inflammatory cytokines *interleukin-6* (*IL-6*) and *IL-8* are key regulators of the inflammatory response during skin aging [45]. Chronic inflammation affects the skin’s structure, promoting protein breakdown and cellular senescence, which results in a loss of elasticity and the appearance of wrinkles [46]. Modulating *IL-6* and *IL-8* is an important strategy to prevent or delay skin aging [46]. Therefore, the expression levels of *IL-6* and *IL-8* in isoschaftoside-treated senescent cells were measured. Isoschaftoside significantly reduced the expression levels of *IL-6* and *IL-8* in senescent cells (Figure 4D,E). These results suggest that inflammatory responses in the skin can be mitigated by reducing inflammatory mediator molecules. Taken together, these findings support the immune defense of the skin by reducing the expression of inflammatory factors through isoschaftoside treatment.

### 2.5. Confirmation of the Senescence Amelioration Gene through Gene Expression Profiling

**Figure 5:**
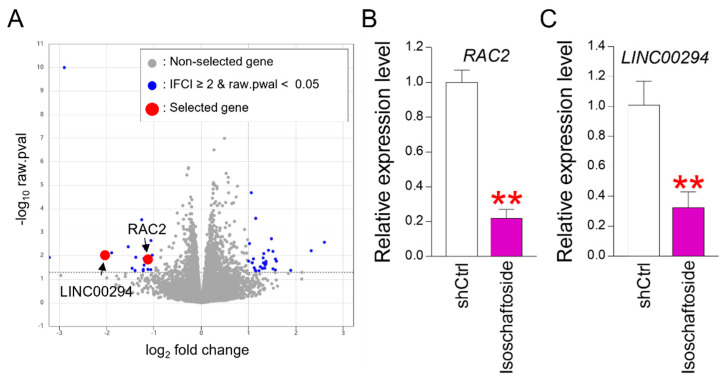
Identification of key regulators of isoschaftoside-mediated senescence amelioration in transcriptome analysis. (**A**) Transcriptome analysis was performed to identify key regulators of isoschaftoside-mediated senescence amelioration. Total RNA sequencing was performed on DMSO- and isoschaftoside-treated senescent cells to compare the overall gene expression levels. Among the significantly regulated genes (blue dots), *RAC2* and *LINC00294* (red dots) were significantly downregulated compared with the DMSO group. The expression analysis of the transcripts was performed in biological triplicates. (**B**) After senescent cells were treated with isoschaftoside, the *RAC2* gene expression level was measured by qPCR (the qPCR primers used are listed in Table 2). Isoschaftoside significantly downregulated *RAC2* in senescent cells. ** *p* < 0.01, Student’s *t*-test. Mean ± SD, n = 3. (**C**) After treatment of senescent cells with isoschaftoside, the level of *LINC00294* gene expression was measured by qPCR (the qPCR primers used are listed in Table 2). Isoschaftoside significantly reduced *LINC00294* in senescent cells. ** *p* < 0.01, Student’s *t*-test. Mean ± SD, n = 3.

This study suggests that isoschaftoside is effective in improving cellular senescence. To investigate the mechanism underlying the senescence amelioration effect mediated by isoschaftoside, we performed gene expression profiling. The profiling compared the DMSO group with the isoschaftoside-treated group. As a result, 51 genes were identified as significantly upregulated or downregulated in isoschaftoside-treated senescent cells (Table S1). Rac family small GTPase 2 (RAC2), a gene, belongs to the Ras superfamily of small GTP-metabolizing proteins [47]. *RAC2* localizes to the plasma membrane and regulates various processes such as secretion, phagocytosis, and cell polarization [47]. The activity of *RAC2* is recognized to play a role in the production of ROS [47]. Long intergenic non-protein-coding RNA 294 (*LINC00294*) is an RNA gene belonging to the lncRNA class [48]. Silencing *LINC00294* is known to reverse the hypoxic inhibition of mitochondrial function [48]. Therefore, we selected *RAC2* and *LINC00294*, which were significantly downregulated, from the gene expression profiles (Figure 5A). qPCR was performed to confirm the significant downregulation of *RAC2* and *LINC00294* identified in the gene expression profiling. *RAC2* and *LINC00294* were significantly decreased in senescent cells upon isoschaftoside treatment (Figure 5B,C). These data support the gene expression profiling results.

**Figure 6:**
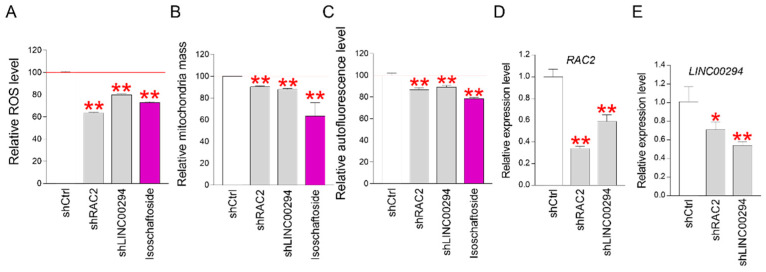
Downregulation of *RAC2* and *LINC00294* genes restores the senescent phenotype. (**A**) ROS of non-knockdown senescent cells (shCTRL), *RAC2* knockdown senescent cells (sh*RAC2*), and *LINC22094* knockdown senescent cells (sh*LINC00294*) were compared. Significant ROS inhibition was observed in sh*RAC2* and sh*LINC00294*. ** *p* < 0.01, Student’s *t*-test. Mean ± SD, n = 3. (**B**) Mitochondrial masses of shCTRL, sh*RAC2*, and sh*LINC00294* were compared. Significant mitochondrial mass reduction was observed in sh*RAC2* and sh*LINC00294*. ** *p* < 0.01, Student’s *t*-test. Mean ± SD, n = 3. (**C**) The lipofuscin (autofluorescence) of shCTRL, sh*RAC2*, and sh*LINC00294* was compared. A significant decrease in autofluorescence was observed in sh*RAC2* and sh*LINC00294*. ** *p* < 0.01, Student’s *t*-test. Mean ± SD, n = 3. (**D**) The expression levels of *RAC2* gene in shCTRL, sh*RAC2*, and sh*LINC00294* were compared by qPCR (the qPCR primers used are listed in Table 2). The expression of *RAC2* was significantly reduced in the sh*RAC2* and sh*LINC00294* groups. ** *p* < 0.01, Student’s *t*-test. Mean ± SD, n = 3. (**E**) The expression levels of *LINC00294* gene in shCTRL, sh*RAC2*, and sh*LINC00294* were compared by qPCR (the qPCR primers used are listed in Table 2). The expression of *LINC00294* was significantly reduced in the sh*RAC2* and sh*LINC00294* groups. * *p* < 0.05, ** *p* < 0.01, Student’s *t*-test. Mean ± SD, n = 3.

The senescence amelioration effects of *RAC2* and *LINC00294*, identified through gene expression profiling, were further investigated. Several studies suggest ROS as a key indicator causing cellular senescence [49]. Therefore, ROS levels in senescent cells were measured using FACS. A significant reduction in ROS was observed in *RAC2* (shRAC2) and *LINC00294* (shLINC00294) knockdown senescent cells, similar to the reduction seen with isoschaftoside treatment (Figure 6A). These data indicate that the downregulation of *RAC2* and *LINC00294* effectively reduces ROS in senescent cells. Reduced ROS mediates the restoration of mitochondrial function and reduces mitochondrial mass [50]. Mitochondrial mass in *RAC2* and *LINC00294* knockdown senescent cells was significantly reduced, comparable to isoschaftoside treatment (Figure 6B), suggesting that *RAC2* and *LINC00294* downregulation restores mitochondrial function. In this study, lipofuscin levels were confirmed as one of the senescence amelioration indicators of isoschaftoside. Lipofuscin levels were significantly reduced in *RAC2* and *LINC00294* knockdown senescent cells, similar to the effect of isoschaftoside (Figure 6C). These results imply that the downregulation of *RAC2* and *LINC00294* may ameliorate senescence.

To investigate the correlation between *RAC2* and *LINC00294*, gene expression analyses were performed on *RAC2* and *LINC00294* knockdown senescent cells. *RAC2* expression was significantly reduced in *LINC00294* knockdown cell lines, and vice versa (Figure 6D,E). These data indicate that the expressions of *RAC2* and *LINC00294* are closely related and that the combined downregulation of these two genes contributes to senescence amelioration.

## 3. Discussion

Previous studies have reported that flavonoids possess diverse structures and a wide range of physiological and biological activities [51]. Astragalin is a flavonoid with kaempferol as its core structure [52]. Isoschaftoside, based on the apigenin structure, has two glucose molecules attached [53]. Skullcapflavone II is also based on apigenin but contains four methoxy groups [54]. 2′-hydroxygenistein is derived from the genistein structure with an additional hydroxyl group [29]. All four flavonoids have been found to provide anti-inflammatory properties via decreasing the expression of cytokines and enzymes [23,25,27,29]. Some flavonoids have antioxidant properties, which may help mitigate the cell damage associated with senescence [24,26]. This study involved an assessment of the ROS-inhibiting effects of these four flavonoids to assess their anti-senescence potential at the cellular level. The screening results indicated that among the four flavonoids, astragalin and isoschaftoside demonstrated significant ROS-inhibiting effects. Both astragalin and isoschaftoside exhibit distinct structural features compared to skullcapflavone II and 2′-hydroxygenistein. Despite being flavonoids with methoxy and hydroxyl group substituents, skullcapflavone II and 2′-hydroxygenistein did not show ROS inhibitory effects. In contrast, astragalin (with glucose as a substituent) and isoschaftoside (with glucose and arabinose as substituents) possess glycoside properties. Our findings suggest that glycoside flavonoids are effective in inhibiting ROS, and their ROS inhibitory effects may be enhanced depending on the number or type of sugar molecules attached. Previous studies have reported that different forms of quercetin induce different absorption and reactions, resulting in different results [55]. Additionally, other previous studies have reported that antioxidant activity varies depending on the structure of flavonoids [56]. Our claim is supported by previous studies showing that activity varies depending on the number and type of sugar molecules attached to the flavonoid moiety.

The C–C glycosidic bond is generally stronger than the C–O bond [57]. Molecules with strong bonds are known to be electrically stable and exhibit reduced electron-donating ability [58]. Reduced electron-donating ability leads to weaker reactions with ROS [59]. However, our results showed that, contrary to established knowledge, the antioxidant activity of molecules with glycosidic bonds (astragalin and isoschaftoside) was significantly higher than that of molecules with methoxy groups (skullcapflavone II and 2′-hydroxygenistein). This observation can be explained by several hypotheses. First, the antioxidant mechanism of flavonoids with methoxy groups may increase the overall electron density of the molecule, thereby enhancing its reaction with ROS. On the other hand, flavonoids with sugar residues may directly react with ROS through the electrons of the hydroxyl group. This hypothesis could explain the high antioxidant capacity of flavonoids with glycosidic bonds. The first hypothesis is supported by previous studies demonstrating the high antioxidant capacity of hydroxyl groups [60,61]. Extending this hypothesis could explain why isoschaftoside with two sugar residues has a greater antioxidant capacity than astragalin. Second, because the C–C glycosidic bond is relatively stable, the antioxidant activity within the cell may last longer than that of flavonoids with a methoxy group. The second hypothesis is also supported by previous studies on the persistence of activity in flavonoids with glycosidic functional groups [62]. We acknowledge the limitations of our study and recognize the need for further research on flavonoids in ROS inhibition and anti-senescence.

Isoschaftoside is a natural flavonoid glycoside found in various plants and has been particularly noted for its anti-inflammatory effects [25]. Isoschaftoside has been reported to inhibit cytokines, which are major mediators that induce inflammatory responses [25]. This contributes to reducing inflammatory responses and alleviating pain and swelling at the site of inflammation. In another study, isoschaftoside inhibited lipopolysaccharide-induced inflammatory responses in human macrophages. Despite these findings, previous research has primarily focused on the anti-inflammation effects of isoschaftoside, with limited knowledge about its impact on intracellular metabolic recovery and aging mechanisms. Our study addresses this gap by investigating both the antioxidant properties and the effects of isoschaftoside on mitochondrial function in senescent cells. We confirmed that isoschaftoside not only enhances mitochondrial function but also reduces glycolysis rates in senescent cells. This suggests that isoschaftoside restores mitochondrial function, thereby improving the overall metabolism of senescent cells and supporting more efficient energy production through glycolysis. To the best of our knowledge, this is the first investigation to demonstrate that isoschaftoside restores metabolic mechanisms in senescent cells by improving mitochondrial function. Our findings may have implications for preventing and treating senescence-related diseases, highlighting the potential of isoschaftoside as a therapeutic agent.

Nicotinamide adenine dinucleotide phosphate (NADPH) is a crucial biochemical reducing agent in cells involved in various reduction reactions, including antioxidant defense, fatty acid synthesis, and cholesterol synthesis [63]. NADPH is particularly critical for the prevention of oxidative stress and the preservation of the cellular redox equilibrium [63]. *RAC2*, a member of the Rho family GTPases, is known to activate the NADPH oxidase complex, which in turn promotes the production of ROS [64]. Previous research has shown that inhibiting *RAC2* can delay cellular senescence by reducing ROS levels generated through NADPH oxidase activity [65]. *LINC00294* is a non-coding RNA that can interact with specific proteins and has been suggested to play a role in regulating ROS, although the exact mechanisms are not fully understood. In this study, we investigated whether downregulating *RAC2* and *LINC00294* could effectively inhibit ROS production in senescent cells. Our results demonstrated that significant suppressions of *RAC2* and *LINC00294* expression led to a marked reduction in ROS levels. This suggests that both *RAC2* and *LINC00294* are important regulators of ROS in senescent cells. The downregulation of *RAC2* and *LINC00294* appears to reduce ROS levels through mechanisms related to various senescence-related signaling pathways. This study highlights the potential of *RAC2* and *LINC00294* as novel therapeutic targets for senescence and senescence-related diseases. Specifically, targeting *RAC2* and *LINC00294* may provide a basis for interventions aimed at mitigating senescence processes by suppressing ROS. However, it is important to note that this study is limited to in vitro experiments. Future research should focus on validating these findings in vivo and exploring the specific mechanisms by which *RAC2* and *LINC00294* regulate senescence. Animal models will be essential to confirm the effectiveness of *RAC2* and *LINC00294* inhibition in delaying senescence and its associated pathologies.

## 4. Materials and Methods

### 4.1. Cell Culture

Human dermal fibroblasts (PCS-201-010; ATCC, Manassas, VA, USA) were used in this study. Cells were cultured in Dulbecco’s modified Eagle’s medium containing 25 mM glucose supplemented with 10% fetal bovine serum (35-015-CV; Corning, Corning, NY, USA), 100 U/mL penicillin, and 100 μg/mL streptomycin (SV30079.01; Hyclone, Waltham, MA, USA). Cells were passaged serially at a 1:2 ratio through old passages. The senescent cell population doubling time was over 14 days.

### 4.2. Preparation of Flavonoid Library

The flavonoid library (astragalin, isoschaftoside, skullcapflavone II, and 2′-hydroxygenistein) was provided by Dr. Ki Yong Lee (College of Pharmacy, Korea University). Astargalin, isoschaftoside, and skullcapflavone II were isolated from *O*. *japonicus*, *V*. *yedoensis*, and *S*. *baicalensis*, respectively [66,67,68]. 2′-hydroxygenistein was isolated from *A*. *americana* according to reference [69], and the structure was identified by ^1^H–, ^13^C–NMR. ^1^H–, ^13^C–NMR data are provided in the supplementary information (Figures S1–S3).

### 4.3. Purity Analysis of Isoschaftoside

The purity of the isoschaftoside isolated from *V. yedoensis* was analyzed using a Waters 2695 HPLC system (Agilent Technolgy, Santa Clara, CA, USA) equipped with a degasser, binary pump, and photodiode array detector. A Shiseido CAPCELL PAK C18 MG column (150 × 4.6 mm, 5 µm) was used for analysis and maintained at room temperature. Mobile phase A was water (0.1% formic acid) and mobile phase B was acetonitrile (0.1% formic acid). Gradient elution was carried out as follows: 0–5 min 15% of B, 5–30 min 15–95% of B. The flow rate was 0.6 mL/min and 10 μL of the sample was injected into the HPLC system. The UV chromatogram of isoschaftoside was acquired at 254 nm using a Waters 996 PAD detector and the areas of peaks were integrated. The purity was determined by HPLC–PDA and was shown to be greater than 94% (Figure S4).

### 4.4. Flow Cytometric Analysis of ROS Screening

The flavonoids (astragalin, isoschaftoside, skullcapflavone II, and 2′-hydroxygenistein) were diluted to a final concentration of 4 µM and treated to senescent fibroblasts for 12 days. Then, cells were incubated for 30 min at 37 °C in a medium comprising 30 µM DHR123 (10056-1; Biotium, Fremont, CA, USA) in order to quantify ROS. Then, cells were processed for flow cytometry analysis as previously explained [70]. Cell counts were measured at 5 × 10^3^ per sample.

### 4.5. Cellular Proliferation Assay

In 96-well plates (353072; Falcon, Corning, NY, USA), senescent fibroblasts were cultivated at a density of 1 × 10^3^ cells per well. Isoschaftoside was diluted to a final concentration of 0–4 µM. Twelve days after isoschaftoside treatment, the cellular proliferation assay was performed using an MTT-based assay. The 96 wells that had been treated with reagents were washed twice with PBS (21-031-CVC; Corning). After removing the washed PBS, the EZ-cytox reagent (EZ-5000; DoGenBio, Seoul, Republic of Korea) was diluted at a ratio of 1:10 in culture medium, and 100 μL was dispensed into each well. After incubation for 1 h in a 37 °C, 5% CO_2_ incubator, the absorbance was measured at 460 nm.

### 4.6. Determination of Cell Viability

A Cedex HiRes Analyzer (05650216001; Roche, Basel, Switzerland) was used to examine the viability of the senescent cells treated with DMSO and isoschaftoside.

### 4.7. Flow Cytometric Analysis of Mitochondrial Membrane Potential (MMP), Mitochondrial Mass, Autophagosome Level, and Cellular Lipofuscin Levels

Isoschaftoside was diluted to a final concentration of 1 µM and used to treat senescent fibroblasts for 12 days. To assess MMP, senescent fibroblasts were treated for 30 min at 37 °C with medium containing 0.6 µg/mL JC-10 (ENZ-52305; Enzo Life Sciences, Farmingdale, NY, USA). To assess mitochondrial mass, senescent fibroblasts were treated for 30 min at 37 °C with medium containing 50 nM MitoTracker™ Deep Red FM Dye (M46753; Invitrogen, Waltham, MA, USA). To assess autophagosome level, senescent fibroblasts were treated for 30 min at 37 °C with medium containing CYTO-ID^®^ (ENZ-51031-0050; Enzo Life Sciences). One commonly used method for quantifying lipofuscin is autofluorescence assessment [71,72,73,74]. To assess autofluorescence, senescent fibroblasts were treated for 30 min at 37 °C with medium containing no staining dye. Then, cells were processed for flow cytometry analysis as previously explained [70]. Cell counts were measured at 5 × 10^3^ per sample.

### 4.8. DNA Tail Length Analysis

DNA damage in senescent cells was quantified by DNA tail length. DNA tail length was imaged using a CometAssay Single Cell Gel Electrophoresis Assay Kit (4250-050-K; R&D systems, Minneapolis, MN, USA). The neutral comet assay was measured as per the manufacturer’s protocol. DNA tail length was analyzed in image j (https://imagej.net/ij/index.html, accessed on 30 August 2024).

### 4.9. Analysis of the Extracellular Acidification Rate (ECAR)

A Seahorse XFe96 analyzer (Agilent Technology, Palo Alto, CA, USA) was used according to the manufacturer’s protocol. ECAR was performed with a Seahorse XF Glycolytic Rate Assay Kit (103344-100; Aglient Technology), as previously described [75].

### 4.10. Immunofluorescence

Immunofluorescence sample preparation was performed according to previous research methods [75]. Rabbit anti-LC3B rabbit antibodies (A19665; 1:200 dilution; Abclonal, Boston, MA, USA), anti-OXPHOS cocktail mouse antibodies (ab110411; 1:200 dilution; Abcam, Cambridge, UK) were used as primary antibodies. Alexa Fluor^®^ 488 goat anti-rabbit IgG antibodies (A-11008; 1:200 dilution; Invitrogen) and Alexa Fluor^®^ 647 goat anti-Mouse IgG antibodies (A-28181; 1:200 dilution; Invitrogen) were used as secondary antibodies. The nuclei were then stained with Hoechst33342 (R37606; Invitrogen) and samples were mounted using Fluorescence Mounting Medium (S3023; Agilent Technology). Images were captured using a Carl Zeiss LSM 700 confocal microscope.

### 4.11. Preparation of Complementary DNA (cDNA)

Senescent fibroblasts were treated with isoschaftoside for 12 days. Total RNA was isolated from 1 × 10^6^ cells using an RNase Mini Kit (74104; QIAGEN, Hilden, Germany) according to the manufacturer’s instructions. Total RNA was reverse-transcribed using a DiaStar™ RT Kit (DR22-R10k; SolGent, Seoul, Korea) according to the manufacturer’s instructions. The purity and concentration of the cDNA were determined using a DS-11 Spectrophotometer (DS-11; DeNovix, Wilmington, DE, USA).

### 4.12. Quantitative PCR (qPCR)

qPCR using cDNA was performed as described previously [76]. The qPCR primers used are described in Table 2.

### 4.13. Transcriptome Expression Profiling

The total RNA from senescent cells treated with isoschaftoside and DMSO was prepared for transcriptome expression profiling. Total RNA was prepared using an RNase Mini Kit (74104; QIAGEN) according to the manufacturer’s instructions. The expression of the prepared transcripts was analyzed by Illumina sequencing (paired ends). To improve the analysis results of the raw reads (101 bp) obtained through sequencing, contamination artifacts were removed. The reads with contamination artifacts removed were mapped to the *Homo sapience* (GRCh38, NCBI_109.20200522) genome using HISAT2 (ver. 2.1.0; Jhons Hopkins University Center for Computational Biology, Baltimore, MD, USA), and aligned reads were generated. Transcriptome assembly was performed using String Tie (ver. 2.1.3b; Jhons Hopkins University Center for Computational Biology), using the aligned reads. Gene set enrichment analysis was performed to target differentially expressed genes. The expression analysis of the transcripts was performed in biological triplicates.

### 4.14. shRNA Plasmid Engineering

In this study, the pLK.O1 (#8453; Addgene, Watertown, MA, USA) vector backbone was used as a lentiviral vector. shCTRL used the pLK.O1 backbone vector. shRAC2 used the shRAC2 (1) and shRAC2 (2) primer sets in the pLK.O1 vector. shLINC00294 used the shLINC00294 (1) and shLINC00294 (2) primer sets in the pLK.O1 vector. The primer sets used are listed in Table 3. All plasmid engineering was performed using HiFi DNA Assembly Master Mix (#E2621L; New Egland Biolab, Ipswich, MA, USA), and the detailed steps are described in a previous study.

### 4.15. Lentiviral Production and Infection

HEK 293T cells were transfected with 5 μg shRNA plasmid mixture (shCTRL, shRAC2 and shLINC00294), 2.5 μg PAX2 plasmid, and 2.5 μg VSV.G plasmid using Lipofectamine 2000 (11668019; Invitrogen, Waltham, MA, USA). Viral supernatant was harvested 24 h after transfection. Polybrene (TR-1003-G; 8 μg/mL; Millipore, Burlington, Middlesex County, MA, USA) was added into the virus production media. Virus infection was performed on the senescent cells, as previously described [75].

### 4.16. Statistical Analyses

Statistical analyses were performed using a standard statistical software package (GraphPad Prism 9; San Diego, CA, USA). Student’s *t*-test and two-way ANOVA followed by Bonferroni’s post hoc test were used to determine whether differences were significant. A two-way ANOVA was performed for data analysis with column factors.

## 5. Conclusions

In this study, we identified that glycoside flavonoids, specifically isoschaftoside, effectively inhibit ROS in senescent cells. Isoschaftoside, which features a glycoside structure, significantly downregulated the genes *RAC2* and *LINC00294*, both of which are implicated in ROS production. By reducing ROS levels, isoschaftoside improved mitochondrial function, which in turn restored cellular metabolism and decreased the reliance on glycolysis. The restoration of mitochondrial function and cellular metabolism led to an amelioration of other senescence-related phenotypes, effectively rejuvenating senescent cells. Our findings highlight a novel mechanism by which isoschaftoside mediates ROS suppression and reprograms the metabolism in senescent cells. The novel mechanism will provide a potential therapeutic strategy to combat cellular senescence and a strategy to improve skin aging.

## Figures and Tables

**Table 1:** Flavonoid library used for screening in this study.

Compound Name	Structure	Molecular Formular	Bioactivity
		Molecular Weight (Da)	
Astragalin	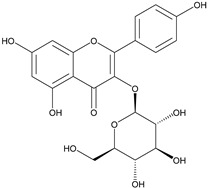	C_21_H_20_O_11_	Anti-inflammation [23] Antioxidants [24]
		448.38	
Isoschaftoside	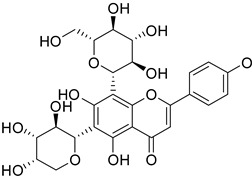	C_26_H_28_O_14_	Anti-inflammation [25]
		564.50	
Skullcapflavone II	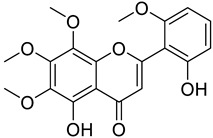	C_19_H_18_O_8_	Antioxidants in brain [26] Anti-inflammation [27]
		374.34	
2′-hydroxygenistein	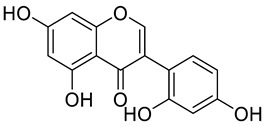	C_15_H_10_O_6_	Anti-melanogenesis [28] Anti-inflammation [29]
		286.24	

**Table 2:** Details of primers used in qPCR.

Target	Orientation	Sequence (5′–3′)	Size (bp)
*36B4*	Forward	CAGCAAGTGGGAAGGTGTAATCC	23
	Reverse	CCCATTCTATCATCAACGGGTACAA	25
*CXCL12*	Forward	TCAGCCTGAGCTACAGATGC	20
	Reverse	CTTTAGCTTCGGGTCAATGC	20
*MMP1*	Forward	ATGAAGCAGCCCAGATGTGGAG	22
	Reverse	TGGTCCACATCTGCTCTTGGCA	22
*COL1A1*	Forward	AGCAAGAACCCCAAGGACAA	20
	Reverse	CGAACTGGAATCCATCGGTC	20
*IL-6*	Forward	CTGATGGGGTCAAATGAAGGTG	22
	Reverse	CGTGCAACCATCCTCCAGAAC	21
*IL-8*	Forward	CTGGCCGTGGCTCTCTTG	18
	Reverse	CCTTGGCAAAACTGCACCTT	20
*RAC2*	Forward	CAACGCTTTCCCGGAGAGT	19
	Reverse	TCCGTCTGTGGATAGGAGAGC	21
*LINC00294*	Forward	TGTGTTGTCCTCCAGAATCG	20
	Reverse	CCAACCAAGAGCCAACAAAG	20

**Table 3:** Details of primers used in shRNA plasmid engineering.

Target	Orientation	Sequence (5′–3′)	Size (bp)
shRAC2 (1)	Forward	GAGGTACTCGAGTACCTCAGGGAACCACTTGGCAATTCTCGACCTCGAGACA	52
	Reverse	CCCTGAGGTACTCGAGTACCTCAGGGAACCACTTGGCCACGCGTGCATACCT	52
shRAC2 (2)	Forward	TTGGAACTCGAGTTCCAAGTACTTGACTGAATCAATTCTCGACCTCGAGACA	52
	Reverse	TACTTGGAACTCGAGTTCCAAGTACTTGACTGAATCCACGCGTGCATACCT	51
shLINC00294 (1)	Forward	GAAATTCTCGAGTTTCCTGATAACTTTGTGGTTAATTCTCGACCTCGAGACA	52
	Reverse	ATCAGGAAACTCGAGAATTTCCTGATAACTTTGTGGCACGCGTGCATACCT	51
shLINC00294 (2)	Forward	TCTATTCTCGAGTAGAGGGTTACATGTTCGCTTAATTCTCGACCTCGAGACA	52
	Reverse	AACCCTCTACTCGAGAATAGAGGGTTACATGTTCGCCACGCGTGCATACCT	51

## Data Availability

Data is contained within the article or Supplementary Material.

## Supplementary Materials

The following supporting information can be downloaded at: https://www.mdpi.com/article/10.3390/molecules29174182/s1, Table S1: Transcriptome analysis list of isoschaftoside-mediated senescent cells. Figure S1. ^1^H–NMR spectrum (600 MHz, CD_3_OD) of 2′-hydroxygenistein isolated from *Apios americana*. Figure S2. ^13^C–NMR spectrum (150 MHz, CD_3_OD) of 2′-hydroxygenistein isolated from *Apios americana*. Figure S3. ESI–MS spectrum of 2′-hydroxygenistein. Figure S4. HPLC chromatogram of isoschaftoside for purity verification.
