# Supplementary material for: Identification of Cellular Isoschaftoside-Mediated Anti-Senescence Mechanism in RAC2 and LINC00294
Source: Molecules. 2024 Sep 4;29(17):4182. doi: 10.3390/molecules29174182 (PMC11397025; doi:10.3390/molecules29174182)
Supplement: Supplementary file 1 [file molecules-29-04182-s001.zip › molecules-3169844-supplementary.pdf]

Supplementary information

Table S1. Transcriptome analysis list of isoschaftoside-mediated senescent cells.

Accession Num.	Gene symbol	Description	Relative expression (Isoschaftoside/DMSO)
NR_046667	ARHGEF7-AS2	ARHGEF7 antisense RNA 2	6.070097
NR_040582	STAG3L3	stromal antigen 3-like 3 (pseudogene)	5.003144
NR_002965	SNORA29	small nucleolar RNA, H/ACA box 29	3.706996
NR_152848	LOC101927759	uncharacterized LOC101927759	3.006354
NR_003934	GTF2IRD1P1	GTF2I repeat domain containing 1 pseudogene 1	2.960729
NR_109828	PCBP2-OT1	PCBP2 overlapping transcript 1	2.956553
NM_021065	H2AC7	H2A clustered histone 7	2.858458
NM_001242672	TTC34	tetratricopeptide repeat domain 34	2.856246
NM_001163079	TMEM121B	transmembrane protein 121B	2.793066
NM_001201	BMP3	bone morphogenetic protein 3	2.674713
NM_001365454	LOC101928764	coiled-coil domain-containing protein 144B	2.577672
XR_002957397	LOC112268089	uncharacterized LOC112268089	2.577227
NM_001289773	MED14OS	MED14 opposite strand	2.543352
NM_001285460	IQGAP2	IQ motif containing GTPase activating protein 2	2.533142
XR_001743969	LOC107986566	uncharacterized LOC107986566	2.497697
NM_001142653	PTGES3L	prostaglandin E synthase 3 like	2.486124
NM_001012337	ROPN1B	rhophilin associated tail protein 1B	2.473032
NM_001206696	IRF6	interferon regulatory factor 6	2.458492
XR_936324	LOC105372275	uncharacterized LOC105372275	2.439507
XR_002957604	LOC101927598	uncharacterized LOC101927598, transcript variant X1	2.371088

NR_110084	LOC101927391	uncharacterized LOC101927391	2.329118
NM_001317837	TMEM155	transmembrane protein 155	2.219330
NM_001077665	AGAP6	ArfGAP with GTPase domain, ankyrin repeat and PH domain 6	2.218167
id-PCDHACT	PCDHACT	protocadherin alpha constant	2.200308
NM_001039592	SPAG8	sperm associated antigen 8	2.172732
XR_923785	LOC105373836	uncharacterized LOC105373836	2.144437
NR_026552	LINC00161	long intergenic non-protein coding RNA 161	2.108917
XM_003959933	LOC101059906	collagen alpha-2(IV) chain-like	2.106612
NR_145459	TALAM1	TALAM1 transcript, MALAT1 antisense RNA	2.079518
NM_001040167	LFNG	LFNG O-fucosylpeptide 3-beta-N-acetylglucosaminyltransferase	2.025174
NM_000748	CHRNB2	cholinergic receptor nicotinic beta 2 subunit	2.000051
NM_001362884	LZTS1	leucine zipper tumor suppressor 1	-2.055557
NM_003259	ICAM5	intercellular adhesion molecule 5	-2.094725
NM_004173	SLC7A4	solute carrier family 7 member 4	-2.100798
NM_002872	RAC2	Rac family small GTPase 2	-2.181698
XR_928234	LOC105375591	uncharacterized LOC105375591	-2.189969
NM_001353493	ASDURF	ASNSD1 upstream reading frame	-2.249973
NM_001033723	ZNF704	zinc finger protein 704	-2.261514
NR_040535	LOC100506472	uncharacterized LOC100506472	-2.317259
NM_001123364	METTL24	methyltransferase like 24	-2.318576
NR_110945	LMF1-AS1	LMF1 antisense RNA 1	-2.343257
NM_004405	DLX2	distal-less homeobox 2	-2.345320

NM_001253845	ADM2	adrenomedullin 2	-2.400193
XR_001744983	LOC105375452	uncharacterized LOC105375452, transcript variant X1	-2.614165
XR_001749292	LOC107984433	uncharacterized LOC107984433	-2.643395
XR_944020	LOC105370560	uncharacterized LOC105370560, transcript variant X1	-2.757979
NM_020459	PAIP2B	poly(A) binding protein interacting protein 2B	-2.920993
NR_037719	TMEM256-PLSCR3	TMEM256-PLSCR3 readthrough (NMD candidate)	-3.724311
NR_015451	LINC00294	long intergenic non-protein coding RNA 294	-4.078606
NM_001195541	TMEM225B	transmembrane protein 225B	-7.450975
NR_037616	BLOC1S5-TXNDC5	BLOC1S5-TXNDC5 readthrough (NMD candidate)	-9.278910

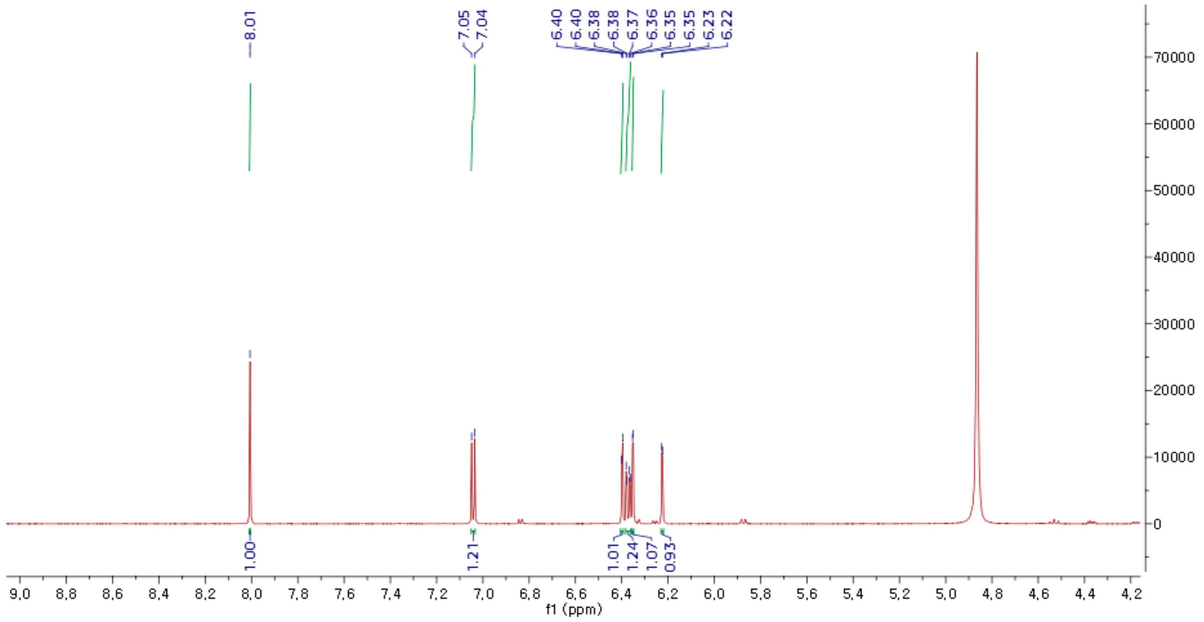

Figure S1. ^{1}H–NMR spectrum (600 MHz, CD_3OD) of 2′-hydroxygenistein isolated from *Apios americana*.

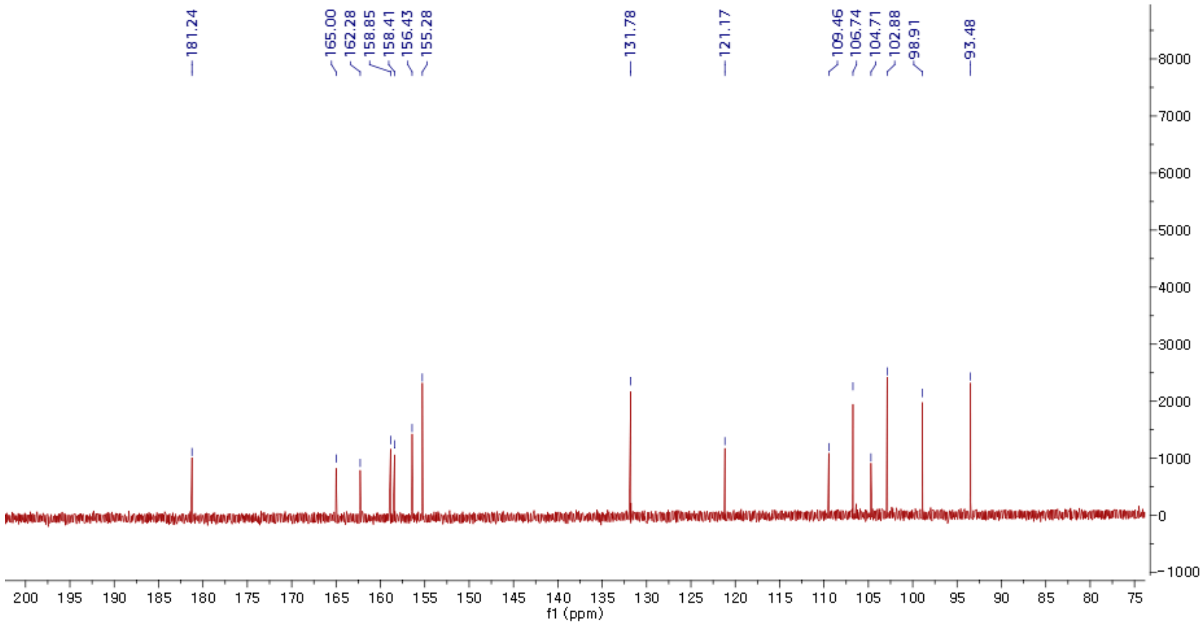

Figure S2. ^{13}C–NMR spectrum (150 MHz, CD_3OD) of 2′-hydroxygenistein isolated from *Apios americana.*

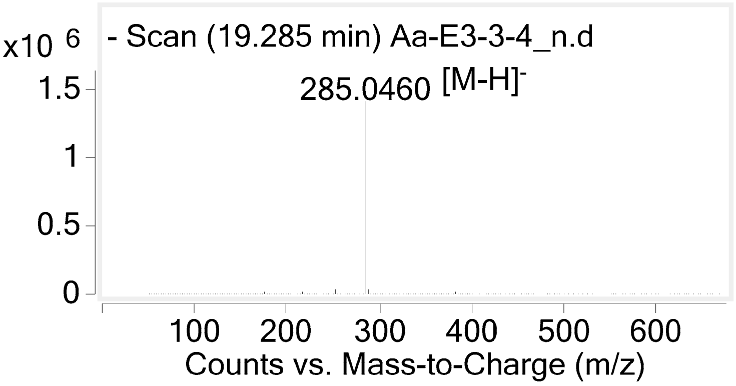

Figure S3. ESI-MS spectrum of 2′-Hydroxygenistein.

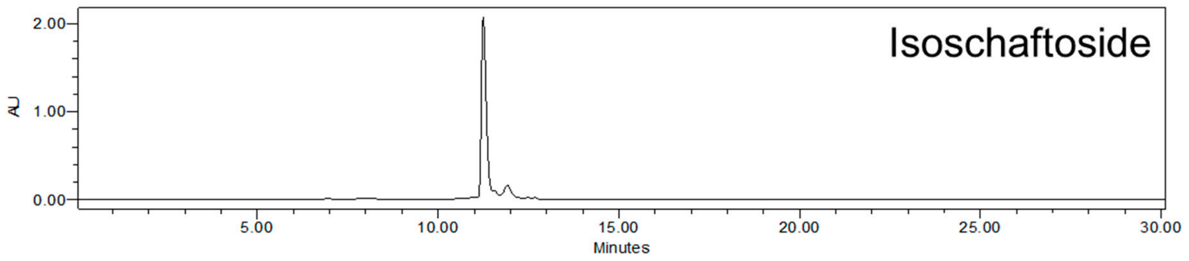

Figure S4. HPLC chromatogram of isoschaftoside for purity verification. The UV chromatogram of isoschaftoside was acquired at 254 nm using Waters 996 PAD detector and the area of peaks were integrated. The purity was determined by HPLC-PDA and was shown to be greater than 94 %.
